# Breast cancer treatment and ethnicity in British Columbia, Canada

**DOI:** 10.1186/1471-2407-10-154

**Published:** 2010-04-21

**Authors:** Parvin Yavari, Maria Cristina Barroetavena, T Greg Hislop, Chris D Bajdik

**Affiliations:** 1 Shahid Beheshti University of Medical Sciences, Tehran, Iran; 2 BC Cancer Agency, Vancouver BC, Canada

## Abstract

**Background:**

Racial and ethnic disparities in breast cancer incidence, stage at diagnosis, survival and mortality are well documented; but few studies have reported on disparities in breast cancer treatment. This paper compares the treatment received by breast cancer patients in British Columbia (BC) for three ethnic groups and three time periods. Values for breast cancer treatments received in the BC general population are provided for reference.

**Methods:**

Information on patients, tumour characteristics and treatment was obtained from BC Cancer Registry (BCCR) and BC Cancer Agency (BCCA) records. Treatment among ethnic groups was analyzed by stage at diagnosis and time period at diagnosis. Differences among the three ethnic groups were tested using chi-square tests, Fisher exact tests and a multivariate logistic model.

**Results:**

There was no significant difference in overall surgery use for stage I and II disease between the ethnic groups, however there were significant differences when surgery with and without radiation were considered separately. These differences did not change significantly with time. Treatment with chemotherapy and hormone therapy did not differ among the minority groups.

**Conclusion:**

The description of treatment differences is the first step to guiding interventions that reduce ethnic disparities. Specific studies need to examine reasons for the observed differences and the influence of culture and beliefs.

## Background

Breast cancer is an important public health issue and major cause of premature mortality in women around the world. Globally, it accounts for 22% of all new cancer diagnoses in women, and approximately 10% of cases in men and women combined. It represents 7% of cancer-related deaths worldwide [1]. In Canada during 2009, breast cancer is estimated to be the most common cancer in women, with more than 22,000 new diagnoses. It is expected to kill more than 5,000 Canadian women in 2009, more than any other type of cancer except lung. One in nine women will be diagnosed with breast cancer during 2009, and 1 in 27 will die from the disease. Breast cancer accounted for an estimated 95,300 potential years of life lost in Canada during 2009 [1].

Canada is becoming more racially and ethnically diverse. Its immigrant population has increased substantially, contributing to growing ethnic communities. Racial and ethnic disparities in breast cancer incidence, stage at diagnosis, survival and mortality are well documented [2]; however few studies have examined disparities in the treatment that breast cancer patients received. It has been reported that African American, Mexican and Puerto Rican breast cancer patients in the United States (US) were less likely to receive the standard of care, whereas Asian and Pacific Islanders were more likely to receive the standard of care [3]. Disparities in the management of early stage breast cancer have been reported among Asian American and Pacific Islanders, particularly among Japanese and Filipinos, and African American breast cancer patients were significantly less likely to receive radiation after lumpectomy than white patients [4,5]. A study in North Carolina found that African American women were less likely to receive hormone therapy and more likely to receive chemotherapy than white women, regardless of the stage at diagnosis [6]. None of these studies reported a decrease in the magnitude of racial disparities between 1992 and 2002.

Treatment decisions may be influenced by many things, including socioeconomic factors, patient-physician interactions, and the patient's beliefs and knowledge. It is the responsibility of physicians to counsel patients about their treatment options, although culture and ethnicity may inhibit this.

Breast cancer is a common cancer in British Columbia (BC) and provincial treatment guidelines have been in effect for several years. This paper compares the treatment received by breast cancer patients for three ethnic groups in BC: Chinese, South Asians and Iranians. These groups correspond to large immigrant communities in the province. Treatment is compared during three time periods: 1980-1989, 1990-1999 and 2000-2006. Values for breast cancer treatments received in the BC general population are provided for reference.

## Methods

All women with histologically-confirmed breast cancer diagnosed between 1980 and 2006 were identified from the population-based BC Cancer Registry (BCCR). It is estimated that more than 95% of all incident cancer cases in BC are registered by the BCCR [7]. We chose 1980 as the starting point for this analysis because immigration among South Asians and Iranians was low before this, and substantial advances in treatment and a provincial treatment guideline were made after 1980. We restricted cases to patients who were referred to the BC Cancer Agency (BCCA) because staging and treatment information was only available in BCCA records. Cases were classified as Chinese, South Asian or Iranian using surname listings. Ethnicity is not recorded in either BCCA or BCCR records. The methodology for classifying ethnicity using surnames has been described in previous papers [8,9]. A total of 3,009 breast cancer cases were analyzed: 1,958 ethnic Chinese, 915 ethnic South Asians and 136 ethnic Iranians.

Information was collected for each case regarding patient characteristics (age at diagnosis, ethnicity and date of diagnosis), tumor characteristics (stage and extent of disease, and tumor size) and primary treatment received (surgery, radiation, chemotherapy and hormone therapy). We separated surgery into two categories depending on whether it was accompanied by radiation. The Tumor, Nodes and Metastasis (TNM) classification of the International Union Contra Cancer (UICC) was used for staging [10].

Information on patient characteristics was obtained from BCCR records that were compiled for a separate research program on cross cultural issues. Information on tumor characteristics and treatment was obtained from BCCA records.

Frequency distributions and proportions of an ethnic group receiving each treatment were calculated according to breast cancer stage. Treatments received by cases were analyzed both together and separately for each diagnosis time period (1980-1989, 1990-1999 and 2000-2006). Differences between ethnic groups in the treatments received were tested using chi-squared statistics. Results for the general population are shown for comparison. The general population includes the ethnic groups, and thus values are not statistically independent from them. Further, unlike values for the ethnic groups, the general population values incorporate breast cancer patients who were not referred to the BCCA for treatment. This also limits formal comparisons involving the general population. The persistence of ethnic disparities over time (i.e., the interaction of ethnic group and time period) was analyzed using a multivariate logistic model. Analyses were conducted using SPSS (version 17), declaring statistical significance when the p-value from a two-sided test was less than 5%.

We received approval for the study from the UBC BCCA Research Ethics Board. (UBC is the University of British Columbia.)

## Results

Basic characteristics of the study groups are shown in Table 1. Age at diagnosis was not significantly associated with ethnicity, although Iranian women tended to be diagnosed at an earlier age than women of the two other ethnicities. Further, 42.6%, 42.1% and 38.3% of Iranian, Chinese and South Asian women were diagnosed before age 50 years. There were no significant ethnic group differences for time period of diagnosis, but there was a very significant difference in the distribution of stage for the three ethnic groups. Higher proportions of Chinese and Iranians were diagnosed with stage I disease compared to South Asians. Conversely, higher percentages of South Asians were diagnosed with stage III or IV breast cancer compared to patients in the Chinese and Iranian groups. All of the specific ethnic groups seem to have been diagnosed at an earlier age than the BC general population, but readers are reminded that the general population includes patients who were not referred to the BCCA.

**Table 1:** Number (%) of patients according to basic demographic and tumor characteristics, 1980-2006.

	Ethnic Group				
			
	Chinese (n = 1,958)	South Asian (n = 915)	Iranian (n = 136)	P-value	General Population
Age at diagnosis (yrs)					
Mean ± SD	54.9 ± 13.4	54.1 ± 12.2	52.7 ± 10.5	0.064	59.8 ± 13.5
					
Diagnosis year				0.261	
1980-1989	216 (11.0)	119 (13.0)	11 (8.1)		9,274 (24.6)
1990-1999	805 (41.1)	353 (38.6)	61 (44.9)		14,928 (39.6)
2000-2006	937 (47.9)	443 (48.4)	64 (47.1)		13,478 (35.8)
					
Stage*				<0.001	
I	808 (45.9)	271 (32.6)	52 (43.7)		15,813 (42.0)
II	695 (39.5)	395 (47.5)	53 (44.5)		16,137 (42.8)
III	103 (5.9)	69 (8.3)	6 (5.0)		3,690 (9.8)
IV	153 (8.7)	97 (11.7)	8 (6.7)		2,040 (5.4)

* non-missing values only

**Figure 1:**
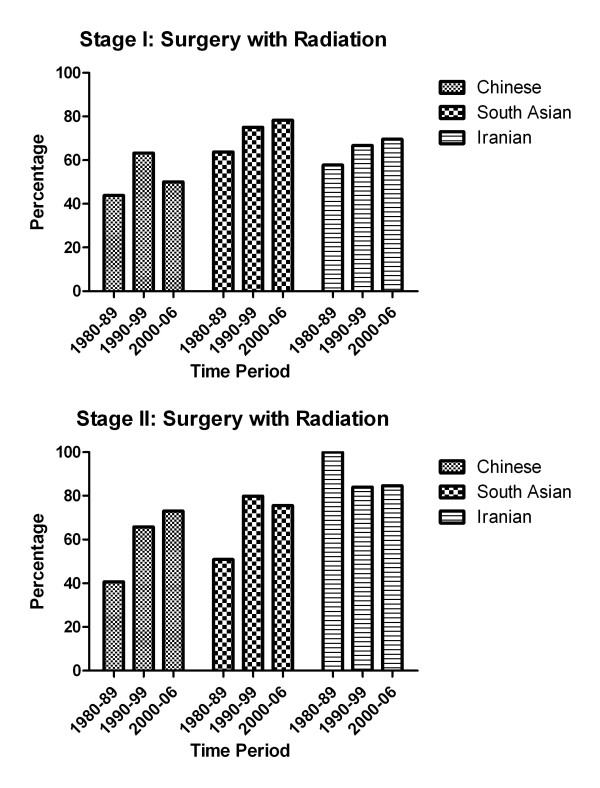
**Proportions of stage I and stage II breast cancer patients in BC that received surgery with radiation, according to ethnic group and time period**.

The types of treatment received among cases from the ethnic groups are shown in Table 2. There were no significant differences in overall surgery use for stage I and II disease among the ethnic groups, however there were significant differences when surgery with and without radiation were considered separately. The appropriateness of radiation usually depends on the type of surgery, so it is difficult to untangle the use of radiation and surgery. Compared to other ethnic groups, Chinese women with stage I or stage II disease were least likely to receive surgery with radiation. Conversely, Chinese women with stage I or stage II disease were most likely to receive surgery without radiation compared to the other ethnic groups. For both surgery with and without radiation, the interaction between ethnicity and time period was not significant at any disease stage. The proportions of stage I and stage II breast cancer patients that received surgery with radiation for each time period is shown in Figure 1. Compared to other ethnic groups, the proportion of Chinese women that received surgery with radiation therapy was lowest during all time periods.

**Table 2:** Number (%) of patients receiving treatment by stage at diagnosis, 1980-2006.

		Ethnic Group				
				
Stage*	Treatment	Chinese	South Asian	Iranian	P-value	General Population
I		n = 808	n = 271	n = 52		n = 15,813
	Radiation	481 (59.5)	191 (70.5)	39 (75.0)	0.001	10,476 (66.2)
	Surgery	787 (97.4)	265 (97.8)	50 (96.2)	0.787	14,981 (94.7)
	Surgery with radiation	477 (59.0)	189 (69.7)	37 (71.2)	0.003	10,266 (64.9)
	Surgery without radiation	310 (38.4)	76 (28.0)	13 (25.0)	0.002	4,715 (29.8)
	Chemotherapy	158 (19.6)	57 (21.0)	9 (17.3)	0.781	2,027 (12.8)
	Hormone therapy	405 (50.1)	115 (42.4)	23 (44.2)	0.077	6,549 (41.4)

II		n = 695	n = 395	n = 53		n = 16,137
	Radiation	468 (67.3)	294 (74.4)	45 (84.9)	0.003	11,163 (69.2)
	Surgery	667 (96.0)	380 (96.2)	53 (100.0)	0.331	14,784 (91.6)
	Surgery with radiation	458 (65.9)	291 (73.7)	45 (84.9)	0.001	10,720 (66.4)
	Surgery without radiation	209 (30.1)	89 (22.5)	8 (15.1)	0.004	4,064 (25.2)
	Chemotherapy	455 (65.5)	260 (65.8)	41 (77.4)	0.208	7,503 (46.5)
	Hormone therapy	408 (58.7)	236 (59.7)	31 (58.5)	0.942	9,363 (58.0)

III		n = 103	n = 69	n = 6		n = 3,690
	Radiation	91 (88.3)	62 (89.9)	6 (100.0)	0.657	3,124 (84.7)
	Surgery	95 (92.2)	64 (92.8)	6 (100.0)	0.777	2,921 (79.2)
	Surgery with radiation	84 (81.6)	60 (87.0)	6 (100.0)	0.355	2,634 (71.4)
	Surgery without radiation	11 (10.7)	4 (5.8)	0 (0.0)	0.397	287 (7.8)
	Chemotherapy	87 (84.5)	57 (82.6)	6 (100.0)	0.531	2,449 (66.4)
	Hormone therapy	75 (72.8)	40 (58.0)	4 (66.7)	0.128	2,199 (59.6)

IV		n = 153	n = 97	n = 8		n = 2,040
	Radiation	119 (77.8)	77 (79.4)	6 (75.0)	0.931	1,437 (70.4)
	Surgery	93 (60.8)	58 (59.8)	4 (50.0)	0.830	878 (43.0)
	Surgery with radiation	79 (51.6)	47 (48.5)	3 (37.5)	0.685	643 (31.5)
	Surgery without radiation	14 (9.2)	11 (11.3)	1 (12.5)	0.832	235 (11.5)
	Chemotherapy	95 (62.1)	59 (60.8)	5 (62.5)	0.979	867 (42.5)
	Hormone therapy	95 (62.1)	62 (63.9)	5 (62.5)	0.958	1,302 (63.8)

* non-missing values only

There were no significant ethnic group differences for other types of treatment at any disease stage. All of the specific ethnic groups seem to have received more chemotherapy than the BC general population for each disease stage, and ethnic groups sometimes seem to have received more hormone therapy than the BC general population. Readers are reminded that the general population includes patients who were not referred to the BCCA.

## Discussion

Breast cancer is predominantly a disease of the economically developed world, but rates are rising rapidly in Asia and economically developing countries. Ethnic differences exist in the distribution of breast cancer histological types, regardless of country of residence. For example, there is a greater incidence of basal type breast cancer within African-American and West African populations [11]. Ethnic differences also exist in breast cancer survival outcomes. An earlier study in BC found that Chinese women had higher breast cancer survival rates than either South Asian women or the female general population. It was speculated that these survival differences may be due to differences in treatment practices and possibly cancer biology because the screening rates were similar among the three population groups [12].

The National Academy of Sciences reported that racial and ethnic minorities generally receive lower quality healthcare than non-minorities [13]. Differences in treatment are known to occur in breast cancer patients of different racial and ethnic groups. A number of factors contribute to this observation, including the types of treatment being offered, the ages of the women examined, and the location of the study. Most studies have compared African American and white women. For example, in some studies breast-conserving surgery was more frequently given to African American women, whereas radiation therapy following breast-conserving surgery was more frequently given to white women [13].

We found that South Asian women with breast cancer tended to be diagnosed at more advanced stages than other women. Previous studies in the US have shown that African Americans, American Indians and Hispanic Whites present with more advanced stages of breast cancer [14-24]. Li and colleagues [3] provided some of the first evidence that Indians and Pakistanis, Mexicans, South and Central Americans, and Puerto Ricans are more likely to present with advanced stages of breast cancer in the US. Poorer survival rates and the more advanced disease stages among these women were attributed to differences in mammography use, obesity and tumor marker expression. Another US study found that differences in staging between African Americans and whites are present among nonusers of mammography but not among regular users [25]. Our study would suggest the need for increased breast cancer screening for South Asian women, so that more cancer cases can be diagnosed at an earlier stage, when treatment is more effective. However, the proportions of women receiving screening mammography in BC who are South Asian or Chinese are similar to those in the BC general population [12].

We found that Chinese women are significantly less likely to receive radiation therapy than South Asian and Iranian women, but this is probably explained by the ethnic groups' differing use of radiation accompanying surgery. The use of chemotherapy or hormone therapy did not differ between these minority groups. An earlier qualitative study examined treatment decision making in a small group of BC Chinese women with *in situ *breast cancer and reported a primary treatment goal was to be rid of their cancer; hence mastectomy was considered to be the treatment of choice [26]. Other treatment options, including breast conserving surgery (which would include radiation), were viewed as less effective options because it would only "control the situation" for a limited period of time [26]. We cannot be certain whether the preference for mastectomy over breast-conserving surgery occurred in our study's patients. A standard treatment protocol for breast cancer patients in BC is determined mainly by the patient's age, type and stage of cancer, tumour sensitivity to certain hormones, and the tumour's expression of the gene HER2. However, patient preference in treatment choice might be contributing to the ethnic differences observed in our study. Future work in examining ethnic group disparities in treatment should not overlook patient preferences.

Other studies also have shown differences in treatments received between ethnic groups. African Americans have been found to be less likely to receive optimal treatment for early or advanced stage disease compared to whites [3]. Cost of treatment may be a contributing factor because African American and Hispanic patients in the US were reported to be more likely to have insurance when compared to non-Hispanic white patients [27]. Some reports suggest no difference in the rates of breast conserving surgery between indigent and well insured populations, whereas other studies suggest that economic status influence the treatment received [27]. However, where there is socialized health care, the economic status of the patient has less influence on treatment delivery. European studies with socialized healthcare report that patients with poorer socioeconomic backgrounds are treated as aggressively as wealthier patients [28]. In the US, patients receiving care within health maintenance organizations, for whom costs are internalized similar to socialized care, may be more motivated to consider downstream costs when making decisions on the initial treatment of breast cancer [29]. In BC, residents receive universal health care coverage and thus costs should not be a determining factor for the choice of treatment.

Recent data suggest that certain patients can omit post-operative radiation therapy after learning the risk and benefits of treatment [27]. Mandelbatt and colleagues have reported that older African American women are twice as likely as white women to have a radiotherapy omitted [30]. Patient's age, stage of disease, co-morbidity/life expectancy, physical functioning and cancer biology all influence the recommendation for chemotherapy. Studies have reported that the likelihood of receiving a recommendation for chemotherapy decreases by 91% per each 10-year age interval [31]. Ethnic groups might include a large proportion of immigrants, and it is unclear whether ethnic groups, and immigrants in particular, have a different likelihood of co-morbidity than others in the population. A difference could explain different use of treatments among the various ethnic groups.

We did not consider survival or other outcomes in this analysis. Survival analyses are often inappropriate in the analysis of retrospective data because of "confounding by indication", where treatments appear to affect survival but actually reflect treatment use that is based on the survival expectation for a patient.

This study had many strengths. Importantly, the data was population-based and abstracted from the provincial cancer registry and the BCCA medical records, both of which have high quality [32]. Essentially all radiotherapy services in BC are provided by the BCCA, as well as a majority of chemotherapy services. The BCCA operates five regional cancer care centers and provides clinics in remote areas. The population of BC is ethnically diverse and well placed for a study of ethnic differences in treatment. The sizes of the three minority groups in this study were large.

There are also limitations to our study. Ethnicity was not recorded in BCCR or BCCA records and had to be determined using surnames. Information about disease stage and treatment was only available for BCCA-referred patients; hence the need to limit the study to these patients. Information about whether a woman received mastectomy or breast-conserving surgery was not available for any patients and this prevents us from determining whether the use of radiation accompanying surgery was appropriate. Another limitation was the lack of information about treatment decision making from both the patient's and physician's perspectives. Our study was a retrospective analysis of patient records in a large cancer registry. The complete rationale that a patient or physician based his or her choice of treatment is not recorded. Finally, we did not consider the extent to which patients completed treatments, although it would be difficult to determine the reasons for this using retrospective data.

## Conclusions

We found that Chinese women with stage I or II breast cancer are significantly less likely to receive surgery with radiation than South Asian and Iranian women. In contrast, Chinese women with stage I or stage II breast cancer are significantly more likely to receive surgery without radiation. Treatment with chemotherapy or hormone therapy did not differ between these groups. Like other studies [5], we found no significant change in ethnic disparities during recent years.

We have reported on differences in treatment received by ethnicity, which has received little attention to date. The description of these differences is the first step to guiding interventions to reduce ethnic disparities. Future studies need to examine reasons for these differences, including the decision making process, culture and beliefs of both the patient and physician.

## Abbreviations

The following abbreviations are used in this paper.

BC: British Columbia; BCCR: BC Cancer Registry; BCCA: BC Cancer Agency; UBC: University of British Columbia; US: United States; TMN: Tumour, Nodes and Metastasis; UICC: International Union Contra Cancer.

## Competing interests

The authors declare that they have no competing interests.

## Authours' contributions

All authors participated in the study's design, data analysis, interpretation and manuscript writing. All authors read and approved the final manuscript.

## Pre-publication history

The pre-publication history for this paper can be accessed here:

http://www.biomedcentral.com/1471-2407/10/154/prepub
